# Evaluating emergency ultrasound training in India

**DOI:** 10.4103/0974-2700.62104

**Published:** 2010

**Authors:** Amit Gupta, Brad Peckler, Michael B Stone, Michael Secko, L R Murmu, Praveen Aggarwal, Sagar Galwankar, Sanjeev Bhoi

**Affiliations:** All India Institute of Medical Sciences, JPN Apex Trauma Center, New Delhi, India; 1 Department of Emergency Medicine, University of South Florida, Tampa General Hospital, Tampa, FL, USA; 2 Department of Emergency Medicine, Kings County Hospital Center/SUNY Downstate, Brooklyn, NY, USA

**Keywords:** Ultrasound, emergency medicine, India, training

## Abstract

**Background:**

In countries with fully developed emergency medicine systems, emergency ultrasound (EUS) plays an important role in the assessment and treatment of critically ill patients.

**Methods:**

The authors sought to introduce EUS to a group of doctors working in the emergency departments (EDs) in India through an intensive 4-day adult and pediatric ultrasound course held at the Apex Trauma Center and EM division of the All India Institute of Medical Sciences in New Delhi. The workshop was evaluated with a survey questionnaire and a hands-on practical test. The questionnaire was designed to assess the current state of EUS in India's EDs, and to identify potential barriers to the incorporation of EUS into current EM practice. The EUS course consisted of a general introductory didactic session followed by pediatric, abdominal and trauma, cardiothoracic, obstetrical and gynecologic, and vascular modules. Each module had a didactic session followed by handson applications with live models and/or simulators. A post-course survey questionnaire was given to the participants, and there was a practical test on the final day of the course. The ultrasound images taken by the participants were digitally recorded, and were subsequently graded for their accuracy by independent observers, residency, and/or fellowship trained in EUS.

**Results:**

There were a total of 42 participants who completed the workshop and took the practical examination; 32 participants filled in the course evaluation survey. Twenty-four (75%) participants had no prior experience with EUS, 5 (16%) had some experience, and 3 (9%) had significant experience. During the practical examination, 38 of 42 participants (90%) were able to identify Morison's pouch on the focused abdominal sonography for trauma (FAST) examination, and 32 (76%) were able to obtain a parasternal long axis cardiac view and identify the left ventricle. The inferior vena cava was identified as it crosses the diaphragm into the right atrium by 20 (48%) participants. All participants felt they would be able to incorporate what they had learned into their practice, and indicated that they were advocates for further training of non-radiologist clinicians in the use of ED ultrasound.

**Conclusion:**

After this introductory workshop in EUS, the participants were comfortable in their ability to use the ultrasound machine. Participants deemed it particularly useful for certain ED applications, particularly the FAST examination, the lung examination, and vascular access.

## INTRODUCTION

India is a developing country which is rapidly evolving and modernizing its emergency medical care. Emergency Medicine (EM) as a distinct academic specialty with residency programs recognized by the Medical Council of India (MCI) does not exist in India, but efforts are being made to initiate such residency programs at various institutions. Currently the emergency departments (EDs) are run by designated casualty medical officers (CMOs), or in a teaching hospital by the senior and junior residents of different specialties such as Internal Medicine, Surgery, and Orthopedics. In countries with fully developed EM systems, emergency ultrasound (EUS) is a critical component of EM practice.[1–5] The use of ultrasound by nonradiologist clinicians is not a new phenomenon in India. The specialties of Obstetrics and Gynecology, Cardiology, and Gastroenterology already use ultrasound as a diagnostic and therapeutic tool, and ultrasound training is an integral part of these residency programs. It is also an ideal resource for the emergency physicians as it is relatively inexpensive, portable, repeatable, delivers no ionizing radiation, and can be used by the treating clinicians immediately at the point of care.[6] In the practice of EM, this reduces the time taken to reach a critical diagnosis as the ultrasound can be done at the bedside by the emergency clinician.[7–11] In India's developing EM system, imaging modalities such as computed tomography (CT), magnetic resonance imaging (MRI), and even traditional technician-performed ultrasound examinations are often not immediately available. EUS helps the treating clinician to diagnose a vast array of emergent conditions immediately at the point-of-care, thus facilitating the rapid diagnosis and treatment of critically ill patients. Introducing emergency clinicians in India to the concept of EUS would, therefore, be of great benefit to the overall quality of care delivered in the ED. The authors sought to introduce EUS to a group of doctors working in EDs in India through a 4-day intensive adult and pediatric ultrasound course held at the Apex Trauma Center and EM division of the All India Institute of Medical Sciences (AIIMS) in New Delhi.

## METHODS

The 4-day EUS course consisted of a general introductory didactic session followed by pediatric, abdominal, trauma, cardiothoracic, obstetrical and gynecologic, vascular, and procedural modules. Each module had an hour-long didactic session followed by 1–2 h hands-on training sessions with live models and/or simulators.

A survey and practical hands-on examination were conducted at the conclusion of the 4-day EUS course, after which approval was obtained from the AIIMS Institutional Review Board.

The post-course survey consisted of 12 questions, primarily focused on participants’ prior ultrasound experience, access to ultrasound equipment, course feedback, and perceived utility and difficulty of individual EUS applications. The practical hands-on examination consisted of three stations where participants were asked to identify specific anatomic structures; the left ventricle as seen on a parasternal long axis view of the heart, a coronal view of Morison's pouch in the right upper quadrant, and a longitudinal view of the inferior vena cava crossing the diaphragm into the right atrium. The ultrasound images taken by the participants were digitally recorded and identified by participant number. These images were subsequently reviewed and graded for their accuracy by independent observers, residency, and/or fellowship trained in EUS.

## RESULTS

**Figure 1:**
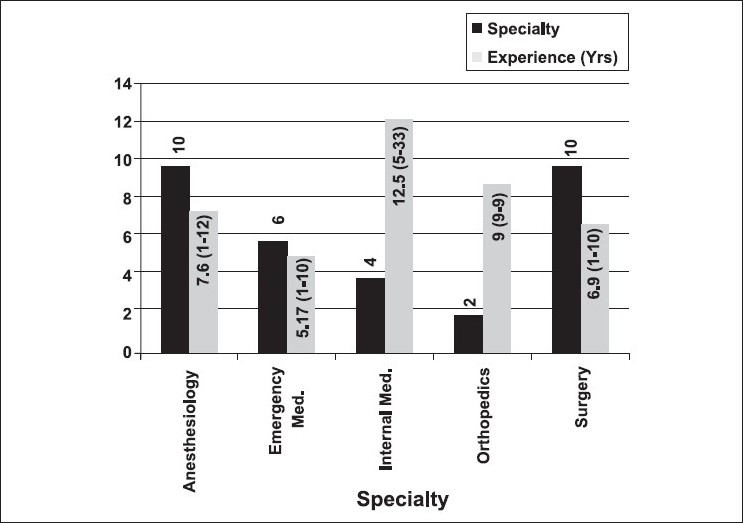
Description of participant specialty with average post residency experience

**Figure 2:**
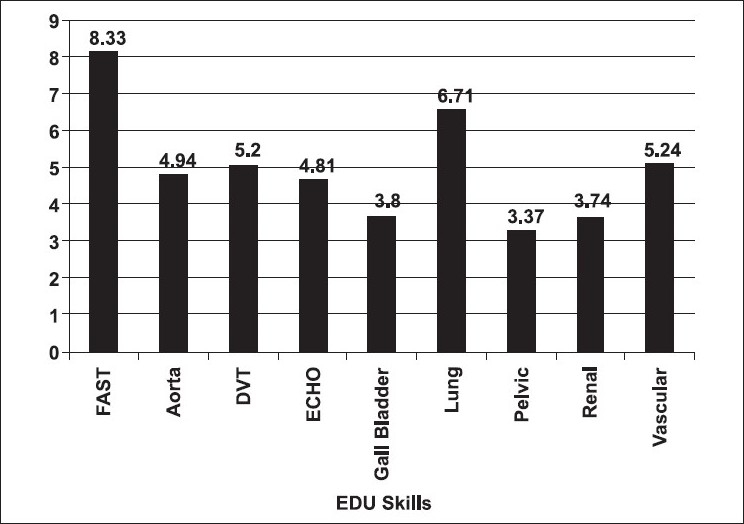
Most useful EDU skill as rated by the participants (1-Least useful to 9-Most useful)

There were 42 participants who completed the EUS course and took the practical examination, while 32 participants completed the course evaluation survey. The specialty distribution and years of practice of the attending participants are given in Figure 1. Twenty-four (75%) participants had no prior experience with EUS, 5 (16%) had some experience, and 3 (9%) had significant experience. Sixteen (50%) of the participants did not have access to an ultrasound machine in their current practice. Eighteen (56%) participants thought that the module on the focused abdominal sonography for trauma (FAST) examination was the easiest and 19 (59%) thought that emergency echocardiography was the hardest to learn. Figure 2 describes what the participants thought about the utility of different ultrasound procedures in their practice. Twenty (63%) participants thought that they would be able to teach this skill in the future. Thirty-one (97%) participants felt that they would have potential access to an ultrasound machine in their practice in the near future.

The results of the practical examination showed that out of a total of 42 participants, 38 (90%) were able to obtain and accurately identify Morison's pouch in the coronal plane, and 32 (76%) participants were able to obtain a parasternal long axis view of the heart and identify the left ventricle. The inferior vena cava was identified by 20 (48%) participants at the point at which it crosses the diaphragm into the right atrium.

All participants felt they would be able to incorporate what they had learned into their practice and likewise indicated that they were advocates for further training of nonradiologist clinicians in the use of EUS.

## DISCUSSION

The participants of the workshop were a highly motivated group of physicians working in the EDs of various hospitals in India. They had received postgraduate training in a variety of different specialties, as there is still no officially recognized EM residency program in India. Given that point-of-care ultrasound is an important part of the curriculum in most EM training programs around the world,[12–15] the authors felt that the EUS workshop would provide an adequate introduction to EUS for practicing emergency physicians as EM residency programs in this country begin to develop.

The survey results suggest that EUS is considered as a highly useful modality by the current practitioners of EM in India, whether anesthesiologists, internists, surgeons, or orthopedists. Course participants from multiple specialties traveled from across the country to attend a 4-day intensive course on EUS as they recognized the enormous potential utility of EUS and wish to incorporate it into their daily practice. The ability to diagnose hemoperitoneum, pneumothorax, cardiac tamponade, deep venous thrombosis, and other life-threatening conditions at the point-of-care using immediately available technology makes EUS an ideal imaging modality for the practicing emergency physicians in India.

The results of the survey and hands-on practical examination demonstrate that this 4-day workshop was perceived as a valuable introduction to the use of ultrasound in EM practice.[16] It is important to note that although 50% of participants have access to an ultrasound machine in the ED, they are currently dependent on the radiologists (who may not be immediately available) to perform and interpret the ultrasound examination. Compared to consultative ultrasound examinations, CT/MRI imagings are often associated with significant delay and expense. The need for focused training and practice in EUS in India is important to help deliver excellent care to the country's critically ill and injured patients.

### Limitations

There is a discrepancy between the number of participants who completed the course and those who filled out the survey forms. As a result, motivated course attendees may have been more likely to participate in the study, and may have performed better than those who did not take the practical examination. The hands-on practical examinations were not comprehensive as participants were asked to perform only a single important component of the total module. Also, hands-on practical examinations were not repeated at a later date to assess participants’ retention of ultrasound skills.

## CONCLUSION

ED clinicians in India were able to perform reasonably well during a practical examination after a focused introductory EUS course. Participants deemed it particularly useful for certain ED applications, specifically the FAST examination, lung examination, and vascular access. With a large percentage of participants reporting the availability of ultrasound in their EDs, adequate training of ED clinicians in EUS may represent the biggest potential barrier to the incorporation of this technology into current medical practice in India.
